# Fishy Business: Effect of Omega-3 Fatty Acids on Zinc Transporters and Free Zinc Availability in Human Neuronal Cells

**DOI:** 10.3390/nu6083245

**Published:** 2014-08-15

**Authors:** Damitha De Mel, Cenk Suphioglu

**Affiliations:** NeuroAllergy Research Laboratory (NARL), School of Life and Environmental Sciences, Faculty of Science, Engineering and Built Environment, 75 Pigdons Road, Waurn Ponds, Victoria 3216, Australia; E-Mail: vdd@deakin.edu.au

**Keywords:** omega-3 fatty acids, DHA, zinc, ZnT3, zinc transporter, free zinc, M17 human neuronal cells, apoptosis

## Abstract

Omega-3 (ω-3) fatty acids are one of the two main families of long chain polyunsaturated fatty acids (PUFA). The main omega-3 fatty acids in the mammalian body are α-linolenic acid (ALA), docosahexaenoic acid (DHA) and eicosapentaenoic acid (EPA). Central nervous tissues of vertebrates are characterized by a high concentration of omega-3 fatty acids. Moreover, in the human brain, DHA is considered as the main structural omega-3 fatty acid, which comprises about 40% of the PUFAs in total. DHA deficiency may be the cause of many disorders such as depression, inability to concentrate, excessive mood swings, anxiety, cardiovascular disease, type 2 diabetes, dry skin and so on. On the other hand, zinc is the most abundant trace metal in the human brain. There are many scientific studies linking zinc, especially excess amounts of free zinc, to cellular death. Neurodegenerative diseases, such as Alzheimer’s disease, are characterized by altered zinc metabolism. Both animal model studies and human cell culture studies have shown a possible link between omega-3 fatty acids, zinc transporter levels and free zinc availability at cellular levels. Many other studies have also suggested a possible omega-3 and zinc effect on neurodegeneration and cellular death. Therefore, in this review, we will examine the effect of omega-3 fatty acids on zinc transporters and the importance of free zinc for human neuronal cells. Moreover, we will evaluate the collective understanding of mechanism(s) for the interaction of these elements in neuronal research and their significance for the diagnosis and treatment of neurodegeneration.

## 1. Introduction

Fish is known as the food for the brain. Although this saying has been around for decades, understanding of the molecular mechanism(s) of fish oil (or more specifically omega-3 fatty acids) in promoting neuronal wellbeing has not been great until recently. With the increase in neurodegenerative diseases globally, many research groups are attempting to gain a better understanding of the molecular detail of this mechanism, and have made some breakthroughs in recent years [1,2,3,4,5,6,7,8]. In this article, we review key findings on the effect of the omega-3 fatty acid DHA on zinc transporters and the importance of free zinc to human neuronal cells. Furthermore, this review article identifies research areas that are in need of further study.

## 2. Fatty Acids

Epidemiological studies have linked high intake of fish and shellfish as part of the daily diet to reduction of the incidence and/or severity of Alzheimer’s disease (AD) and senile mental decline in general [3]. Many studies over the past few decades have shown the nutritional value of fish, particularly in regard to omega-3 fatty acids [4]. Omega-3 fatty acids are one of the two main families of a broader group of fatty acids referred to as polyunsaturated fatty acids (PUFAs). The other main family of PUFAs encompasses the omega-6 fatty acids. In general, PUFAs are essential in many biochemical events, especially in early post-natal development processes such as cellular differentiation, photoreceptor membrane biogenesis and active synaptogenesis [9]. Despite the significance of these two families, mammals cannot synthesize PUFA *de novo*, so they must be ingested from dietary sources. Though belonging to the same family, both omega-3 and omega-6 fatty acids are metabolically and functionally distinct and have opposing physiological effects. In the human body, high concentrations of omega-6 fatty acids are known to increase the formation of prostaglandins and thereby increase inflammatory processes [10]. On the other hand, the reverse process can be seen with increased omega-3 fatty acids in the body. Many other factors, such as thromboxane A2 (TXA2), leukotriene B4 (LTB4), IL-1, IL-6, tumor necrosis factor (TNF) and C-reactive protein, which are implicated in various health conditions, have been shown to be increased with high omega-6 fatty acids but decreased with omega-3 fatty acids in the human body [10].

Dietary fatty acids have been identified as protective factors in coronary heart disease [11], and PUFA levels are also known to play a critical role in immune responses, gene expression and intercellular communications [12]. Mainly, omega-3 fatty acids are known to be vital in the prevention of fatal ventricular arrhythmias [13]. Omega-3 fatty acids are also known to reduce thrombus formation propensity by decreasing platelet aggregation, blood viscosity and fibrinogen levels [14]. Since omega-3 fatty acids are prevalent in the nervous system, it seems logical that a deficiency may result in neuronal problems, and this is indeed what has been identified and reported in the literature [15,16,17,18,19,20]. The main omega-3 fatty acids in the mammalian body are α-linolenic acid (ALA), docosahexenoic acid (DHA) and eicosapentaenoic acid (EPA). In general, seafood is rich in omega-3 fatty acids, more specifically DHA and EPA (Table 1). Thus far, there are nine separate epidemiological studies that suggest a possible link between increased fish consumption and reduced risk of AD [15] and eight out of ten studies have reported a link between higher blood omega-3 levels and reduced cognitive decline [16]. In another study conducted with individuals of 65 years of age or older (*n* = 6158), it was found that only high fish consumption, but not dietary omega-3 acid intake, had a protective effect on cognitive decline [17]. In 2005, following a meta-analysis study of the available epidemiology and preclinical studies, clinical trials were conducted to assess the effects of omega-3 fatty acids on cognitive protection [18]. Four of the trials completed have shown a protective effect of omega-3 fatty acids only among those with mild cognitive impairment conditions [19]. In another trial with 485 subjects with mild memory complaints, an improvement with 900 mg of DHA was demonstrated [20].

**Table 1:** Total percentage of omega-3 fatty acids in common foods and supplements. Table adopted from Maclean C.H. *et al.* [18].

Food/Supplement	EPA	DHA	ALA	Total %
*Fish*				
Salmon	X	X		>50%
Sardine	X	X		>50%
Anchovy	X	X		>50%
Halibut	X	X		>50%
Herring	X	X		>50%
Mackerel	X	X		>50%
Tuna	X	X		>50%
Fresh Bluefin	X	X		>50%
*Oils/Supplements*				
Fish oil capsules	X	X		>50%
Cod liver oils	X	X		>50%
Salmon oil	X	X		>50%
Sardine oil	X	X		>50%
Black currant oil			X	10%–50%
Canola oil			X	10%–50%
Mustard seed oils			X	10%–50%
Soybean oil			X	10%–50%
Walnut oil			X	10%–50%
Wheat germ oil			X	10%–50%
*Seeds and other foods*				
Flaxseeds/Linseeds			X	>50%
Spinach			X	>50%
Wheat germ			X	10%–50%
Human milk			X	10%–50%
Peanut butter			X	<10%
Soybeans			X	<10%
Olive oil			X	<10%
Walnuts			X	<10%

DHA is the most abundant fatty acid in neural membranes, imparting appropriate fluidity and other properties [21], and is thus considered as the most important fatty acid in neuronal studies [8]. DHA is well conserved throughout the mammalian species despite their dietary differences [22]. DHA is mainly concentrated in membrane phospholipids at synapses and in retinal photoreceptors [23] and also in the testis and sperm [22]. In adult rats’ brain, DHA comprises approximately 17% of the total fatty acid weight, and in the retina it is as high as 33% [24]. DHA is believed to have played a major role in the evolution of the modern human and in particular the well-developed brain [25]. Premature babies fed on DHA-rich formula show improvements in vocabulary and motor performance [26]. Analysis of human cadaver brains have shown that people with AD have less DHA in their frontal lobe and hippocampus compared with unaffected individuals [27]. Furthermore, studies in mice have increased support for the protective role of omega-3 fatty acids. Mice administrated with a dietary intake of DHA showed an increase in DHA levels in the hippocampus. Errors in memory were decreased in these mice and they demonstrated reduced peroxide and free radical levels [28], suggesting a role in antioxidant defense. Another study conducted with a Tg2576 mouse model of AD demonstrated that dietary DHA supplementation had a protective effect against reduction in drebrin (actin associated protein), elevated oxidation, and to some extent, apoptosis via decreased caspase activity [29].

## 3. Zinc and Life

Zinc is a trace element, which is indispensable for life, and it is the second most abundant trace element in the body [30]. Zinc is known to be related to growth, development, differentiation, immune response, receptor activity [31], DNA synthesis, gene expression, neuro-transmission, enzymatic catalysis, hormonal storage and release, tissue repair, memory, the visual process [32] and many other cellular functions. Moreover, the indispensability of zinc to the body can be discussed in many other aspects, including as a component of over 300 different enzymes [33] or as an integral component of a metallothioneins [34] or as a gene regulatory protein [35]. Approximately 3% of all proteins contain zinc binding motifs [36]. The broad biological functionality of zinc is thought to be due to its stable chemical and physical properties [37]. Zinc is considered to have three different functions in enzymes; catalytic, coactive and structural [38]. Indeed, it is the only metal found in all six different subclasses of enzymes [39]. The essential nature of zinc to the human body can be clearly displayed by studying the wide range of pathological effects of zinc deficiency. Anorexia, embryonic and post-natal growth retardation, alopecia, skin lesions, difficulties in wound healing, increased hemorrhage tendency and severe reproductive abnormalities, emotional instability, irritability and depression are just some of the detrimental effects of zinc deficiency [31,33,40,41,42,43].

Proper development and function of the central nervous system (CNS) is highly dependent on zinc levels. In the mammalian organs, zinc is mainly concentrated in the brain at around 150 µm [33]. However, free zinc in the mammalian brain is calculated to be around 10 to 20 nm [44,45] and the rest exists in either protein-, enzyme- or nucleotide bound form [31]. The brain and zinc relationship is thought to be mediated through glutamate receptors [30], and zinc is known to inhibit both excitatory and inhibitory receptors [36]. Vesicular localization of zinc in pre-synaptic terminals is a characteristic feature of brain-localized zinc, and its release is dependent on neural activity [30]. Zinc deficiency is also related to many CNS abnormalities. Retardation of the growth and development of CNS tissues have been linked to low zinc levels [38]. Peripheral neuropathy, spina bifida, hydrocephalus, anencephalus [46], epilepsy and Pick’s disease [47] have also been linked to zinc deficiency.

Although zinc deficiency causes much damage to the body, the body cannot tolerate excessive amounts of zinc. In both human and animal cells, neurotoxicity and neurodegeneration are widely seen with high levels of zinc [30,48,49]. Translocation of zinc from pre-synaptic to post-synaptic neurons is the main mechanism thought to be effected in neurotoxicity, epilepsy [47] and brain trauma [50]. The relationship between zinc and neurodegeneration, specifically AD, has been interpreted in several ways. One study has proposed that β-amyloid has a greater propensity to form insoluble amyloid in the presence of high physiological levels of zinc [51]. Insoluble amyloid is thought to aggregate to form plaques, which is a main pathological feature of AD [52]. Further studies have shown that chelation of zinc ions can deform and disaggregate plaques [53]. In AD, the most prominent injuries are found in hippocampal pyramidal neurons, acetylcholine-containing neurons in the basal forebrain, and in somatostatin-containing neurons in the forebrain [30]. All of these neurons are known to favor rapid and direct entry of zinc in high concentrations [54], leaving neurons frequently exposed to high dosages of zinc. This is thought to promote neuronal cell damage through oxidative stress and mitochondrial dysfunction [30].

Excessive levels of zinc are also capable of inhibiting Ca^2+^ and Na^+^ voltage gated channels [55,56,57] and up-regulating the cellular levels of reactive oxygen species (ROS) [58]. Elevated ROS levels are directly detrimental to cell survival by damaging key macromolecules such as nucleic acids, proteins and lipids, which results in rapid cell death. Moreover, high levels of zinc are found in Alzheimer’s brains indicating a possible zinc related neurodegeneration [30]. A study conducted with mouse neuronal cells has shown that even a 24-h exposure to high levels of zinc (40 µm) is sufficient to degenerate cells [59].

Although it is vital to regulate and maintain zinc levels in the human body, assessment of the zinc status is not an easy task [60]. This is mainly due to the lack of a specific and sensitive biochemical index of zinc nutrition. Owing to its indispensable nature, many organisms are capable of maintaining zinc homeostasis despite a low dietary intake. If the human diet is deficient in zinc, the body efficiently conserves zinc at the tissue level by compensating other cellular mechanisms to delay the dietary deficiency effects of zinc. These include reduction of cellular growth rate and zinc excretion levels, and redistribution of available zinc to more zinc dependent cells or organs [61,62]. Plasma or serum zinc is the most commonly used marker to assess the zinc levels, however plasma and serum zinc levels are known to be relatively insensitive to dietary zinc. More recently, a novel method of measuring metallothionein (MT) levels was introduced as a more reliable alternative. Although it shows promise as a biomarker, more research is required to determine the specificity of MT for the assessment of the zinc status of individuals and populations. In humans, erythrocyte metallothionein (E-MT) levels may be considered as a better indicator of zinc depletion and repletion, as E-MT levels are sensitive to dietary zinc intake [60,63,64]. Once again, further research is required to assess E-MT as a specific biomarker for zinc status. However, it should be noted here that MT plays an important role in zinc homeostasis by acting as a target for zinc ion binding and thus assisting in the trafficking of zinc ions through the cell, which may be similar to that of zinc transporters as discussed in Section 4 below.

## 4. Zinc Transporters

As discussed in Section 3 above, deficient or excess amounts of zinc in the body can be catastrophic to the integrity of cellular biochemical and biological systems, indicating the necessity for an appropriate and sophisticated homeostatic mechanism. Primarily, this is achieved by the gastrointestinal system controlling the absorption, excretion and the distribution of zinc [65], although the hydrophilic and high-charge molecular characteristics of zinc are not favorable for passive diffusion across the cell membranes [66]. Thus, in most, if not in all of these mechanisms, zinc movement is known to occur via intermembrane proteins and zinc transporter (ZnT) proteins [67]. These transporters are mainly categorized under two metal transporter families; Zip (ZRT, IRT like proteins) and CDF/ZnT (Cation Diffusion Facilitator) [67]. Both families are also known as SLC (Solute Linked Carrier) gene families: Zip (SLC-39) and ZnT (SLC-30) [68]. More than 20 zinc transporters have been identified and characterized over the last two decades (14 Zips and 8 ZnTs) [67,68].

**Figure 1:**
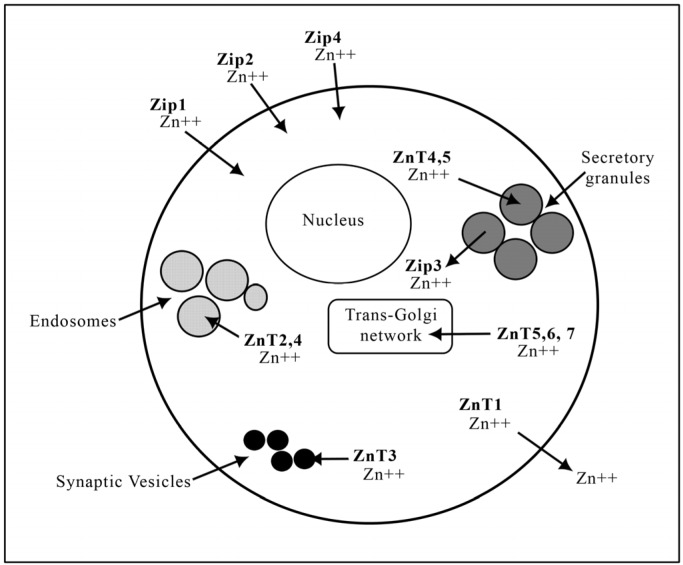
Putative cellular localization of some of the different human zinc transporters (*i.e.*, Zip1- Zip4 and ZnT1- ZnT7). Arrows indicate the direction of zinc passage by the appropriate putative zinc transporters in a generalized human cell. Although there are fourteen Zips and eight ZnTs known so far, only the main zinc transporters are illustrated in this figure for clarity and brevity.

Members of the SLC39 family have been identified as the putative facilitators of zinc influx into the cytosol, either from the extracellular environment or from intracellular compartments (Figure 1). The identification of this transporter family was a result of gene sequencing of known Zip1 protein transporters in plants, yeast and human cells [69]. In contrast to the SLC39 family, the SLC30 family facilitates the opposite process, namely zinc efflux from the cytosol to the extracellular environment or into luminal compartments such as secretory granules, endosomes and synaptic vesicles; thus decreasing intracellular zinc availability (Figure 1) [70]. Despite the significant increase in the knowledge of these transporters over recent years, many aspects of the molecular mechanisms of zinc metabolism are yet to be understood. Out of all of the zinc transporters, ZnT3 is the most important in the brain where it is responsible for the transport of zinc into the synaptic vesicles of glutamatergic neurons in the hippocampus and neocortex, which will be discussed in detail in Section 5 below.

## 5. DHA and Zinc Homeostasis

Many studies have identified possible associations between DHA levels, zinc homeostasis, neuroprotection and neurodegeneration. Dietary DHA deficiency resulted in increased zinc levels in the hippocampus and elevated expression of the putative zinc transporter, ZnT3, in the rat brain [71]. In this study, rats were raised under control diets, which were either omega-3 enriched or DHA-deficient. Rats raised under the deficient diet demonstrated an up-regulation of ZnT3 mRNA levels and free zinc in the hippocampus. Although this study reports an increase in ZnT3 mRNA levels, it lacks protein data to assess the functionality or availability of the ZnT3 protein since changes at the RNA level do not necessarily equate to changes at the protein expression level. In addition, altered zinc metabolism in neuronal cells has been linked to neurodegenerative conditions such as AD [30]. Another study conducted with transgenic mice has shown a significant link between ZnT3 transporter levels and cerebral amyloid plaque pathology. When the ZnT3 transporter was silenced in transgenic mice expressing cerebral amyloid plaque pathology, a significant reduction in plaque load and the presence of insoluble amyloid were observed [72]. In addition to the decrease in plaque load, ZnT3 silenced mice also exhibited a significant reduction in free zinc availability in the hippocampus and cerebral cortex. Collectively, the findings from this study are very interesting and indicate a clear connection between zinc availability and amyloid plaque formation, thus indicating a possible link to AD. DHA supplementation has also been reported to limit the following: amyloid presence, synaptic marker loss, hyper-phosphorylation of Tau, oxidative damage and cognitive deficits in transgenic mouse model of AD [73]. In addition, studies by Stoltenberg, Flinn and colleagues report on the modulation of zinc and the effect in transgenic mouse models of AD [74,75]. Given that all of these are classic pathological features of AD, and considering the limiting nature of DHA in these processes, it can be argued that DHA is a key candidate in preventing or even curing this debilitating disease.

In order to better understand the possible links and pathways of zinc and DHA with neurodegeneration, we designed a study that incorporates all three of these aspects, to study their effects at the cellular level. In this study, we were able to demonstrate a possible link between omega-3 fatty acid (DHA) concentration, zinc availability and zinc transporter expression levels in cultured human neuronal cells [7]. When treated with DHA over 48 h, ZnT3 levels were markedly reduced in the human neuroblastoma M17 cell line. Moreover, in the same study, we were able to propose a possible neuroprotective mechanism of DHA, which we believe is exerted through a reduction in cellular zinc levels (through altering zinc transporter expression levels) that in turn inhibits apoptosis [7]. DHA supplemented M17 cells also showed a marked depletion of zinc uptake (up to 30%) [7], and free zinc levels in the cytosol were significantly low compared to the control treatment (unpublished data). This reduction in free zinc availability was specific to DHA; cells treated with EPA had no significant change in free zinc levels (unpublished data). Moreover, DHA-repleted cells had low levels of active caspase-3 and high Bcl-2 levels compared to the control treatment [7]. These findings are consistent with previous published data and further strengthen the possible correlation between zinc, DHA and neurodegeneration [30,71,72,73,74,75]. On the other hand, recent studies using ZnT3 knockout (ZnT3KO) mice have shown the importance of ZnT3 in memory and AD pathology [76,77,78]. For example, Sindreu and colleagues have used ZnT3KO mice to establish the important role of ZnT3 in zinc homeostasis that modulate presynaptic MAPK signaling required for hippocampus-dependent memory [76]. In another study using the ZnT3KO mice, it has been shown that ablation of ZnT3 may represent a phenocopy for the synaptic and memory deficits of AD [77]. Finally, with the use of the ZnT3KO mice, Martel and colleagues have shown that ZnT3 is involved in memory and behavior dependent on the hippocampus and perirhinal cortex [78]. Collectively, all of these three ZnT3KO mice studies indicate the important role of ZnT3 in memory [76,77,78]. However, further research is required to establish the relevance of such mouse model findings to the human system.

**Figure 2:**
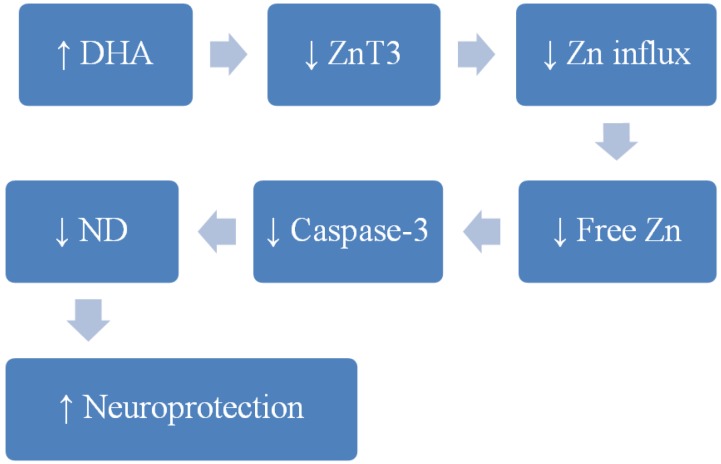
Proposed neuroprotection mechanism of docosahexaenoic acid (DHA) in reference to synaptic zinc. Schematic diagram showing possible benefits of DHA in neuroprotection through reduction of ZnT3 transporter expression levels in human neuronal cells, which results in a reduction of zinc flux and thus lowering zinc concentrations in neuronal synaptic vesicles, and therefore contributing to a lower incidence of neurodegenerative diseases (ND), such as Alzheimer’s disease (AD).

As discussed above, due to the importance of zinc, especially labile zinc in cells and tissues, it is vital to have a comprehensive understanding of the concentration, distribution, localization, kinetics and functions of free zinc. Moreover, in a therapeutic sense, it will be beneficial to understand and develop mechanisms where the freely available zinc pool can be regulated. However, the mechanism by which DHA and zinc interact or effect neuronal cell survival is yet to be identified; although recent studies using cultured human neuronal cells (including studies from our research group), have been able to shed some light on this matter, as discussed above. Results from these studies indicate a possible zinc-transporter-expression-level-dependent mechanism for DHA neuroprotection. Collectively from these studies, the following possible mechanism can be proposed (Figure 2).

More recent data from our research group have also shown a link between the expression levels of histone H3 and H4 proteins in human neuronal cells in relation to DHA and zinc. Following DHA treatment, both H3 and H4 levels were up-regulated. In contrast, zinc treatment resulted in a down-regulation of histone levels [6]. Both zinc and DHA have shown opposing effects on histone post-translational modifications, indicating a possible distinctive epigenetic pattern [4]. Upon treatment with zinc, M17 cells displayed an increase in histone deacetylase (HDACs) and a reduction in histone acetylation. Conversely, with DHA treatment, HDAC levels were significantly reduced and the acetylation of histones was up-regulated [4]. These findings also support a possible interaction between DHA and zinc availability.

## 6. Conclusions and Future Perspectives

Considering the large body of reported data and findings, it is possible to safely claim that there is more than one potential pathway by which DHA and zinc interact at a cellular level, at least in cultured human neuronal cells. Significance and importance of both DHA and zinc in neuronal survival is attested by the presence of these multiple mechanisms. Thus, this highlights the importance of greater molecular understanding of the subject matter and the need for further investigation. Most of these reported studies were conducted using human neuroblastoma cells, or similar cell types, due to the lack of live mature human neuronal cells. Thus, the results may differ from results achieved under actual human physiological conditions due to the structural and functional differences between these cells and mature human neurons. Therefore, an alternative approach that can mimic the human neuronal cells more effectively would be advantageous. For example, the use of differentiated neuroblastoma cells instead of undifferentiated cells might help to give a better and more appropriate insight into the discussed molecular mechanisms.
